# Transcriptome analysis reveals changes in lignin and flavonoid biosynthesis in *Serendipita indica* colonized Tartary buckwheat

**DOI:** 10.3389/fpls.2025.1595781

**Published:** 2025-06-05

**Authors:** Wenjing Wang, Shanpu Zhong, Wuyao Tang, Xingmei Zhou, Shengjie Li, Binhan Ding, Tao Wang, Tongliang Bu, Zizhong Tang, Qingfeng Li

**Affiliations:** ^1^ College of Life Sciences, Sichuan Agricultural University, Ya’an, China; ^2^ College of Modern Technology, Mianyang City College, Mianyang, China

**Keywords:** Tartary buckwheat, *Serendipita indica*, RNA-seq, lignin biosynthesis, flavonoid biosynthesis, phenylpropanoid biosynthesis

## Abstract

**Introduction:**

Tartary buckwheat (*Fagopyrum tataricum* Gaertn.), classified as a food and herbal medicinal crop, offers substantial nutritional benefits but suffers from poor yields and quality. Studies indicate that *Serendipita indica* positively impacts Tartary buckwheat's yield and quality, yet the underlying processes remain largely unexplored.

**Methods:**

This study aimed to examine the genetic transcript of Tartary buckwheat in both colonized and uncolonized *S. indica*.

**Results:**

It was discovered that the pathway for producing phenylpropanoids in Tartary buckwheat, both in colonized and uncolonized *S. indica*, both in colonized and uncolonized *S. indica*, was found to be enriched in KEGG (Kyoto Encyclopedia of Genes and Genomes). Genetic expression analysis of lignin and flavonoid biosynthesis pathways in colonized *S. indica* showed a comparison between lignin biosynthesis pathway genes in colonized *S. indica* and those in uncolonized *S. indica* in Tartary buckwheat. Research revealed a decrease in certain genes linked to lignin synthesis and an increase in others associated with flavonoid biosynthesis in both colonized and uncolonized *S. indica* Tartary buckwheat. Furthermore, research revealed a reduction in lignin levels in Tartary buckwheat stems and seeds both colonized and uncolonized by *S. indica*, in contrast to an increase in flavonoid levels in leaves and seeds of Tartary buckwheat colonized and uncolonized by the same fungi.

**Discussion:**

Findings indicate that the process of synthesizing lignin and flavonoids could offer valuable insights into how *S. indica* enhances Tartary buckwheat's yield and quality.

## 1 Introduction

Tartary buckwheat is an annual herb of the Polygonaceae buckwheat. Tartary buckwheat, recognized as a cash crop, boasts significant nutritional content and resembles authentic grains (Giménez-Bastida and Zieliński, 2015). Its composition includes flavonoids (Kim et al., 2007; Kalinova and Vrchotova, 2009), starch (Giménez-Bastida and Zieliński, 2015), raw protein (Janovská et al., 2021) and trace elements (Aubert et al., 2021; Kasar et al., 2021) along with various other healthful compounds. Consequently, Tartary buckwheat has gained favor in the global market and is considered a strategic crop that can provide human nutrition (Chettry and Chrungoo, 2021). Nonetheless, the production and quality of Tartary buckwheat face distinct challenges. The cultivation of Tartary buckwheat often occurs in regions with elevated temperatures, arid conditions, and barren soils, which affect its growth and lead to reduced crop yields (Liu et al., 2025). Concurrently, the challenge of dehulling Tartary buckwheat in the industry lies in its complexity. While steam dehulling aids in this process, its drawbacks include being time-intensive, expensive, and diminishing the quality of Tartary buckwheat, which fails to meet the substantial market demand (Wang and Campbell, 2007). Consequently, enhancing the production and quality of Tartary buckwheat has become a significant challenge that must be addressed. Presently, certain endophytic fungi are known to enhance plant nutrient absorption and growth by improving nutrient absorption efficiency, acclimatizing the plant to its growing conditions and maintaining plant health, thereby fostering growth and increasing yield (Almario et al., 2017).

The endophytic fungus *Serendipita indica*, extracted from the soil of the Indian desert, establishes itself in plant roots (Verma et al., 1998; Varma et al., 1999). The fungus *Serendipita indica* is a member of the *Sebacinaceae* family, classified under the Basidiomycota division (Weiss et al., 2004; Li et al., 2023a). *S. indica* offers numerous advantages in facilitating nutrient absorption by plants, resisting diseases, enduring stress, and enhancing growth, and is commonly used in agricultural and ecological fields (Li et al., 2023a). It has been shown that *S. indica* promotes the growth of *Cerasus humilis* plants and enhances their antioxidant enzymes by increasing the indole acetic acid (IAA) content of the plants, which in turn improves the quality of *Cerasus humilis* (Yin et al., 2024). Extensive research has demonstrated that symbiotic colonization by *S. indica* enhances abiotic stress resilience in diverse plant species, including *Hordeum vulgare* L (Waller et al., 2005), *Centella asiatica* (Jisha et al., 2018), *Passiflora edulis* (Yan et al., 2021) and Tartary buckwheat (Zheng et al., 2023). This mutualistic association not only improves plant growth parameters but also elevates secondary metabolite production, while concurrently strengthening both abiotic stress toleranceand biotic stress resistance against phytopathogens. Tartary buckwheat roots can be colonized by *S. indica* in abiotic stress conditions, and via phytohormone metabolism in Tartary buckwheat, it enhances both its growth and that of the plant. The build-up of soluble sugars, proteins, flavonoids, and phenolics has been shown to increase Tartary buckwheat’s abiotic stress (Zheng et al., 2023). Nevertheless, it remains uncertain how *S. indica* enhances Tartary buckwheat production and bolsters its ability to withstand stress.

Here, we hypothesized that *S. indica* has a promoting effect on Tartary buckwheat yield and quality. To explore this possibility, we investigated the effect of *S. indica* on Tartary buckwheat in the absence of stress through a transcriptomic approach. This study deepens *S. indica*’s understanding of the regulatory mechanisms of lignin and flavonoid biosynthesis in Tartary buckwheat, and provides theoretical guidance for improving Tartary buckwheat yield and quality.

## 2 Materials and methods

### 2.1 Plant materials and fungi preparation

The seeds were immersed in water at a temperature of 42°C for 2 hours. Full-sized samples were sterilized using a 0.1% mercuric chloride solution for 10 minutes before use (Sasidharan and Jayachitra, 2017). *S. indica* was inoculated in solid complete medium (CM) and maintained at 28°C for two weeks. Additionally, *S. indica*, measuring 7.5 mm, was introduced into 200 mL of CM liquid medium and kept at 28°C and 120 rpm for 20 days. Subsequently, 2 g of mycelium were gathered and combined with 100 mL of sterilized double-distilled water to achieve a 2% (w/v) (± 1.5 × 10^7^ spores/mL) *S. indica* suspension (Abdelaziz et al., 2019). The soils used for potting trials were obtained from Ya’an, China(29°58’N, 102°59’E), where one batch of soil was inoculated with *S. indica* liquid culture at 100 mL/kg named P group, while a separate batch received an identical volume of distilled water for control purposes named CK group. Each containing 8 replicate pots (12 cm diameter × 10cm height) with four Tartary buckwheat plants per pot. Cultivation occurred under natural photoperiod conditions (11/13 h light/dark cycle) with average daytime temperature maintained at 20 ± 5°C and watered every two days. Roots, stems, leaves and seeds of Tartary buckwheat were collected after the 40th day of cultivation.

### 2.2 Colonization of *S. indica* in roots and measurement of plant growth indices

After 15 days of co-cultivation, collection of roots of colonized and uncolonized Tartary buckwheat plants, soaked in 10% KOH for 30 min, then in 1% HCl for 20 min, then stained with 0.02% Trypan Blue solution for 2 h, and finally decolorized with 50% lactophenol solution for 2 h (Rai et al., 2001). The colonization of *S. indica* in Tartary buckwheat roots was observed with an Olympus fluorescence microscope, BX53 (Olympus, Shanghai, China). *S. indica* colonization in buckwheat roots was observed under an Olympus fluorescence microscope BX53 (Olympus, Shanghai, China). Next, colonized and uncolonized Tartary buckwheat plants (three samples per group) were selected and weighed using an MTQ300 electronic scale (Mellen, Shenzhen, China) for weighing the fresh weight of the above-ground and below-ground parts of Tartary buckwheat. The plant samples were then dried in an oven at 60°C until constant weight, and the dry weight of the plants was weighed using an MTQ300 electronic scale (Meilun, Shenzhen, China).

### 2.3 Determination of total lignin and flavonoid content

After about 40 days of co-cultivation, 4 plants were taken from each pot to determine the lignin and the flavonoid content. To determine the lignin content of *S. indica* colonized and uncolonized Tartary buckwheat, the samples were dried in an oven until constant weight. After passing through a 35-mesh sieve, 3 mg of the sample was weighed, and the lignin content of Tartary buckwheat was determined using a lignin content detection kit (Beijing Box Biotechnology Co., Ltd., Beijing, China) and an enzyme labeling instrument, Multiskan SkyHigh 500C (ThermoFisher Scientific, Shanghai, China). Each replicate was performed three times. To determine the flavonoid content of *S. indica* colonized and uncolonized Tartary buckwheat, the samples were dried in an oven at 60°C until constant weight. After passing through a 35-mesh sieve, 0.1 g of the sample was weighed and extracted by ultrasonic extraction at 300 w, 60°C for 30 min. The supernatant was collected by centrifugation at 12000 rpm, 25°C for 10 min. The flavonoid content of Tartary buckwheat was determined using a Plant Flavonoid Content Assay Kit (Micro method) (Beijing Solabao Technology Co., Ltd., Beijing, China) and an enzyme labeling instrument, Multiskan SkyHigh 500C (ThermoFisher Scientific, Shanghai, China). Each replicate was performed three times.

### 2.4 Lignin staining

The paraffin sections were routinely dewaxed and hydrated, then stained with Senna (1% prepared in ultrapure water) for 1 to 2 h; washed slightly under running water; and then stained with solid green (1% prepared in 95% ethanol) for 30–60 s. The sections were then stained with anhydrous ethanol I for 2 min, anhydrous ethanol II for 2 min and anhydrous ethanol II for 2 min. Then they were sequentially dehydrated by anhydrous ethanol I for 2 min and anhydrous ethanol II for 2 min, then by xylene I for 1 min and xylene II for 2 min, and finally sealed with neutral gum and examined microscopically (Brandizzi, 2000).

### 2.5 RNA isolation, library construction and sequencing

Roots of colonized and uncolonized Tartary buckwheat were collected and a total of 18 samples were collected. Three biological replicates were made for each treatment group. Total RNA was extracted from the plants, and the integrity and total amount of RNA were accurately detected using an Agilent 2100 bioanalyzer. After RNA extraction, purification and library construction, the libraries were subjected to paired-end (PE) sequencing using Next-Generation Sequencing (NGS) based on the Illumina sequencing platform. The transcriptome sequencing was performed by Suzhou PANOMIX Biomedical Tech Co. DEGs were identified based on a fold change (FC) >1 and a false discovery rate (FDR) value below 0.05.

### 2.6 Quantitative real-time PCR analysis

To validate the lignin-flavonoid-related pathway, key enzyme genes were selected and primers were designed for further analysis (**Supplementary Table S1**). Total RNA was extracted in fresh plant samples using a total RNA isolation kit (Vazyme, Nanjing, China). Total RNA (1 µg) was reverse-transcribed into first-strand cDNA using the HiScript III RT SuperMix for qPCR (+gDNA wiper) kit (Vazyme, Nanjing, China).Genes were amplified using the ChamQ Universal SYBR qPCR Master Mix kit (Vazyme, Nanjing, China) using gene-specific primers according to the manufacturer’s instructions. QPCR amplification was performed under the following thermal cycling conditions: initial denaturation at 95°C for 30 s, followed by 40 cycles of denaturation at 95°C for 10 s and annealing/extension at 60°C for 30 s. Gene expression was calculated by using the 2^-ΔΔCT^ method. To normalize the gene transcript levels, the *FtH3* gene was co-amplified as a reference gene. Three biological replicates were done for each sample.

### 2.7 Data analysis

The graphs in the paper were plotted and statistically calculated using origin 2021 software. Student’s t-test was performed using IBM SPSS Statistics 27 program to test the significance of the data obtained, a significance threshold of P < 0.05. Data are means and standard deviations of three independent biological replicates.

## 3 Results

### 3.1 Differences in phenotypes of Tartary buckwheat colonized by *S. indica*

**Figure 1:**
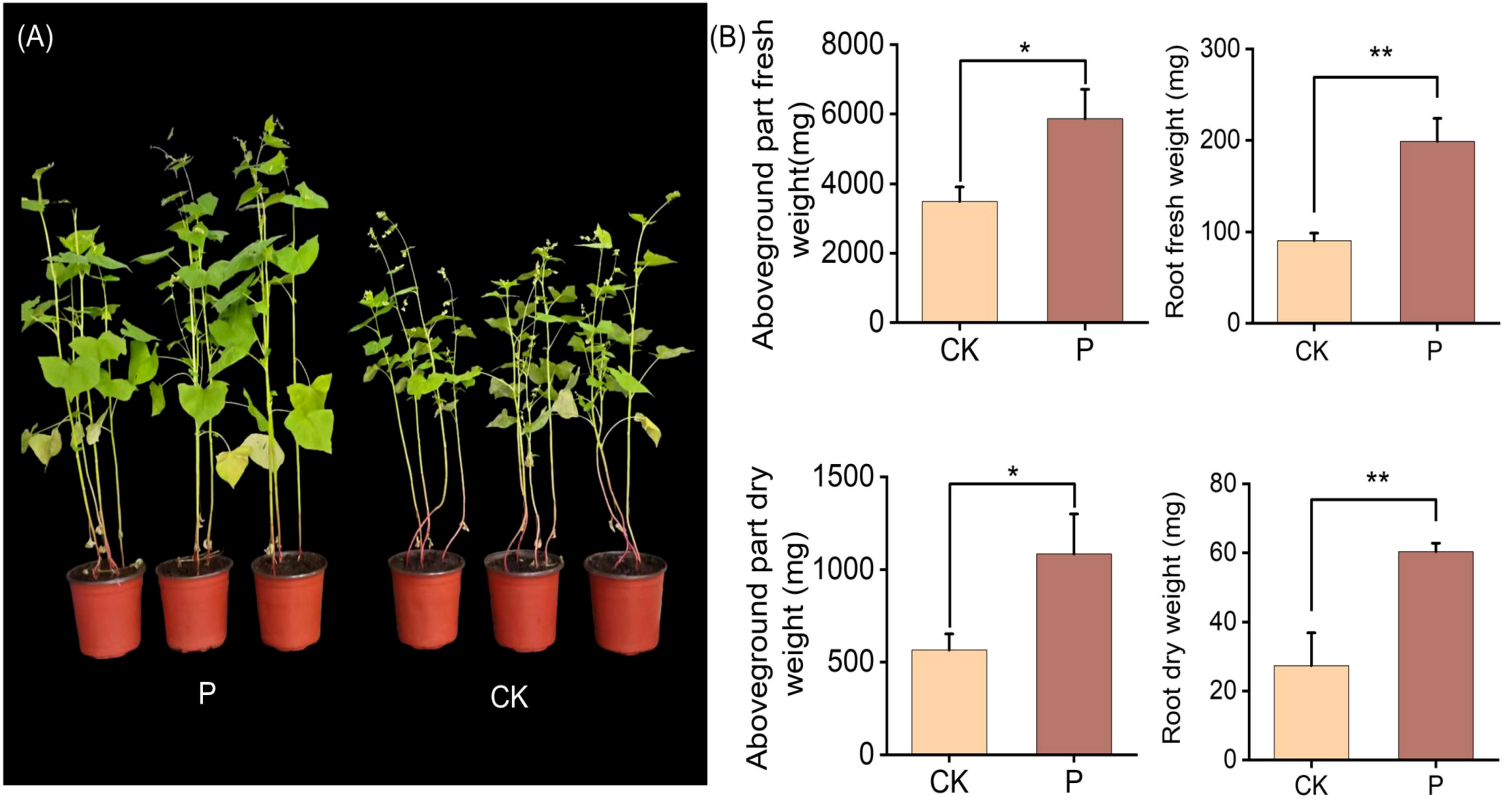
Analyzing plant phenotypes of Tartary buckwheat after inoculation with *S. indica*. **(A)** Control (CK) and *S. indica* colonized (P) Tartary buckwheat plants; **(B)** Fresh and dry weights of Aboveground parts and Root of CK and P Tartary buckwheat plants. Values are expressed as mean ± SD (standard deviation) of three replicates. *p < 0.05; **p < 0.01.

Tartary buckwheat in colonized *S. indica* had a significant change from that of uncolonized *S. indica*, with a tendency for larger leaves and thicker rhizomes in colonized *S. indica* (**Figure 1A**; **Supplementary Figures S1**, **S2**). *S. indica* led to a significant increase in significant increase in aboveground part and root biomass of Tartary buckwheat, where the aboveground part and root fresh weight increased by 1.68 and 2.20 times, and the aboveground part and root dry weight increased by 1.91 and 2.21 times (**Figure 1B**). The results showed that there was a significant difference between Tartary buckwheat colonized with *S. indica* and uncolonized with *S. indica*, and *S. indica* significantly increased the biomass of Tartary buckwheat.

### 3.2 Overall RNA-seq results and quality control in Tartary buckwheat colonized by *S. indica*

**Figure 2:**
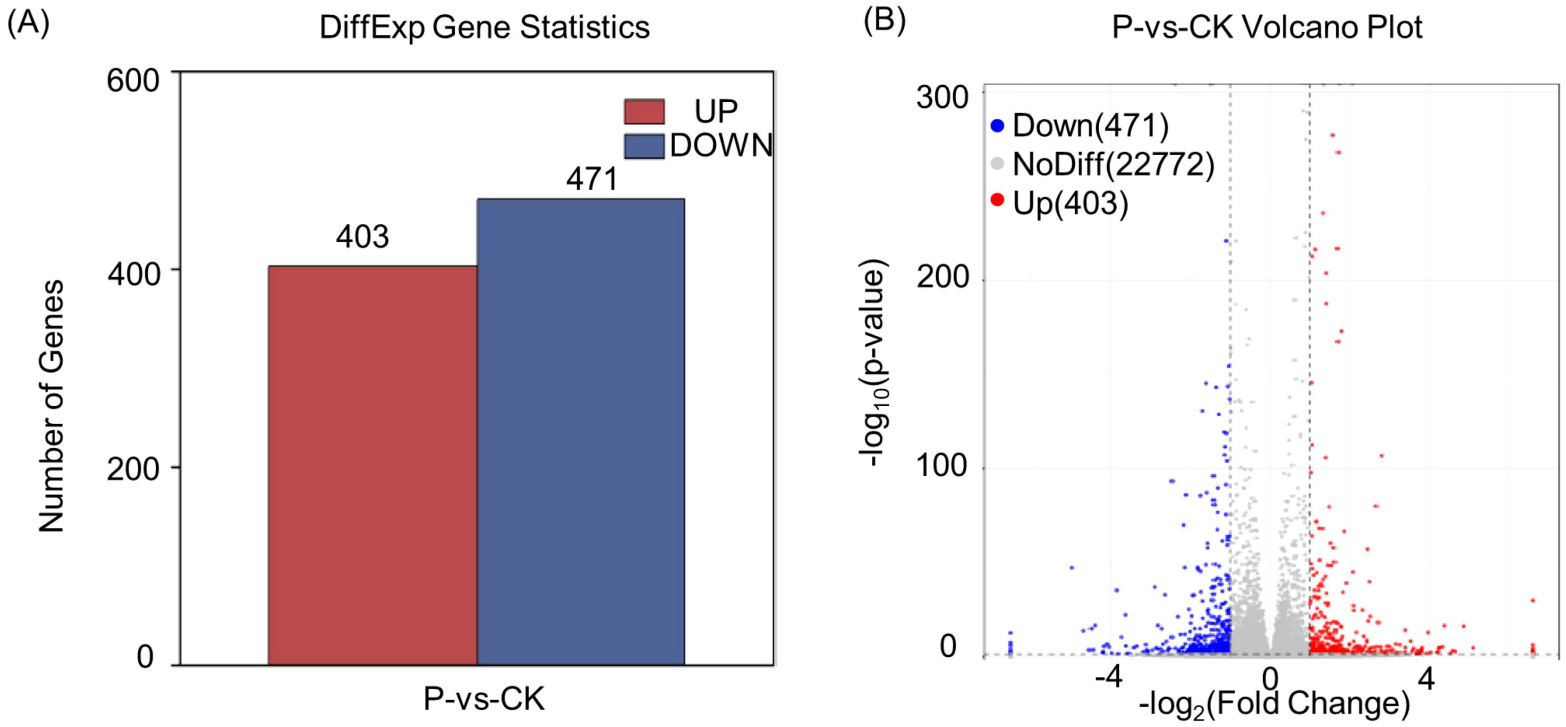
Differentially expressed gene statistics **(A)** and the volcano plot of P vs CK **(B)**. P: *S. indica* colonized Tartary buckwheat plants and CK: *S. indica* uncolonized Tartary buckwheat plants.

**Figure 3:**
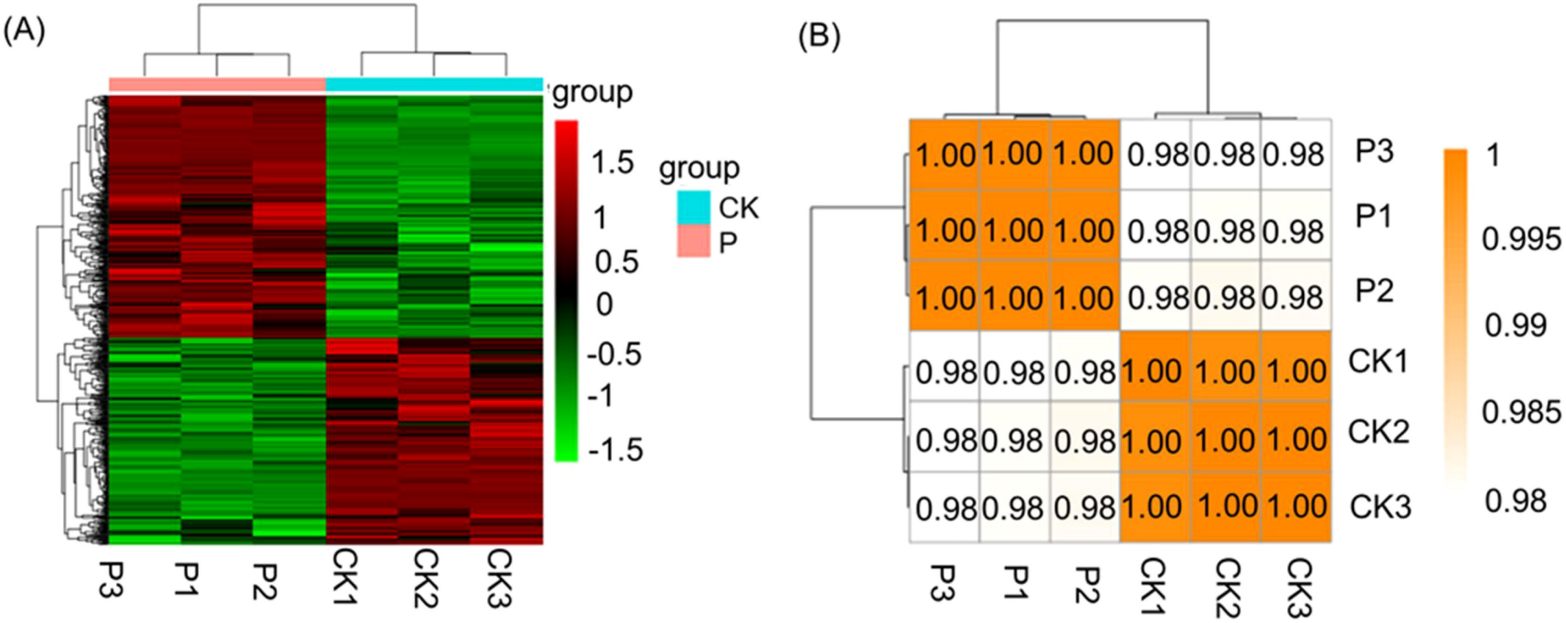
Sample correlation heatmap **(A)** and Comparison group of P vs CK heatmap **(B)**. **(A)** The red color shows the number of up regulated genes, and the green color shows the number of down regulated genes. P: *S. indica* colonized Tartary buckwheat plants and CK: *S. indica* uncolonized Tartary buckwheat plants.

In order to explore the regulatory mechanisms of *S. indica* on Tartary buckwheat in terms of growth promotion and resistance, the transcriptomes of the roots of Tartary buckwheat with significant phenotypic differences (divided into two groups, each group was repeated three times) were selected for sequencing. Total RNA was extracted for transcriptome sequencing and gene expression profiling. The statistics of RNA sequencing (RNA-seq) reads for each sample are shown in **Supplementary Table S2**. These six libraries yielded a total of 39,306,277,876 raw reads from Illumina sequencing, with an average of 6,551,046,313 reads per sample. After quality control filtering, these six libraries yielded a total of 38,691, 425,229 clean reads, with an average of 6,448,570,872 clean reads per sample. the average quality scores of clean reads, Q20 and Q30, were 98.72% and 96.27%, respectively, indicating that the clean reads obtained were of high quality. The differentially expressed genes (DEGs) statistics of Tartary buckwheat are presented in **Figure 2A**. It is evident that 403 genes were significantly up-regulated and 471 genes were significantly down-regulated (log2 (fc)) by the addition of *S. indica* treatment. The volcano plot (**Figure 2B**) depicted the gene expression pattern by revealing the number of genes that were up-regulated, down-regulated and those that did not undergo significant changes. Further, the reliability of the data was determined by correlation analysis between the samples, and as shown in **Figure 3A**, the correlation coefficients (R^2^) between the groups were above 98%, indicating high correlation between the groups and reliable data. In addition, hierarchical clustering of DEG patterns was represented by heatmaps to present the clustering results (**Figure 3B**). Genes with similar expression patterns may have common functions or participate in common metabolic and signaling pathways.

### 3.3 Functional enrichment analysis of DEGs

**Figure 4:**
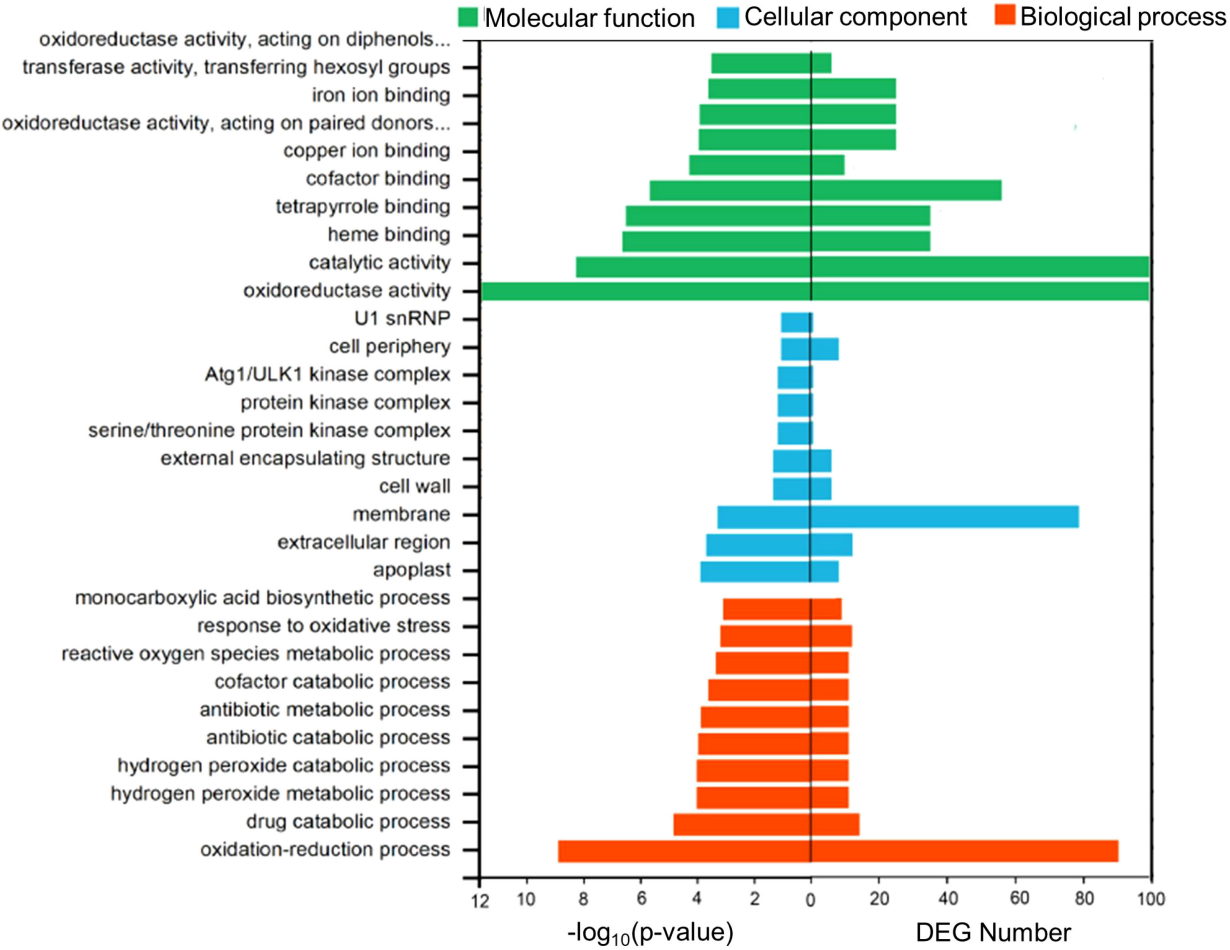
Histogram of the GO enrichment. Analyses with the top 10 entries with the smallest p-value selected for each Biological Process (BP), Cellular Component (CC), and Molecular Function (MF). The left histogram is the -log_10_ (p-value) and the right histogram is the DEGs number of the corresponding.

**Figure 5:**
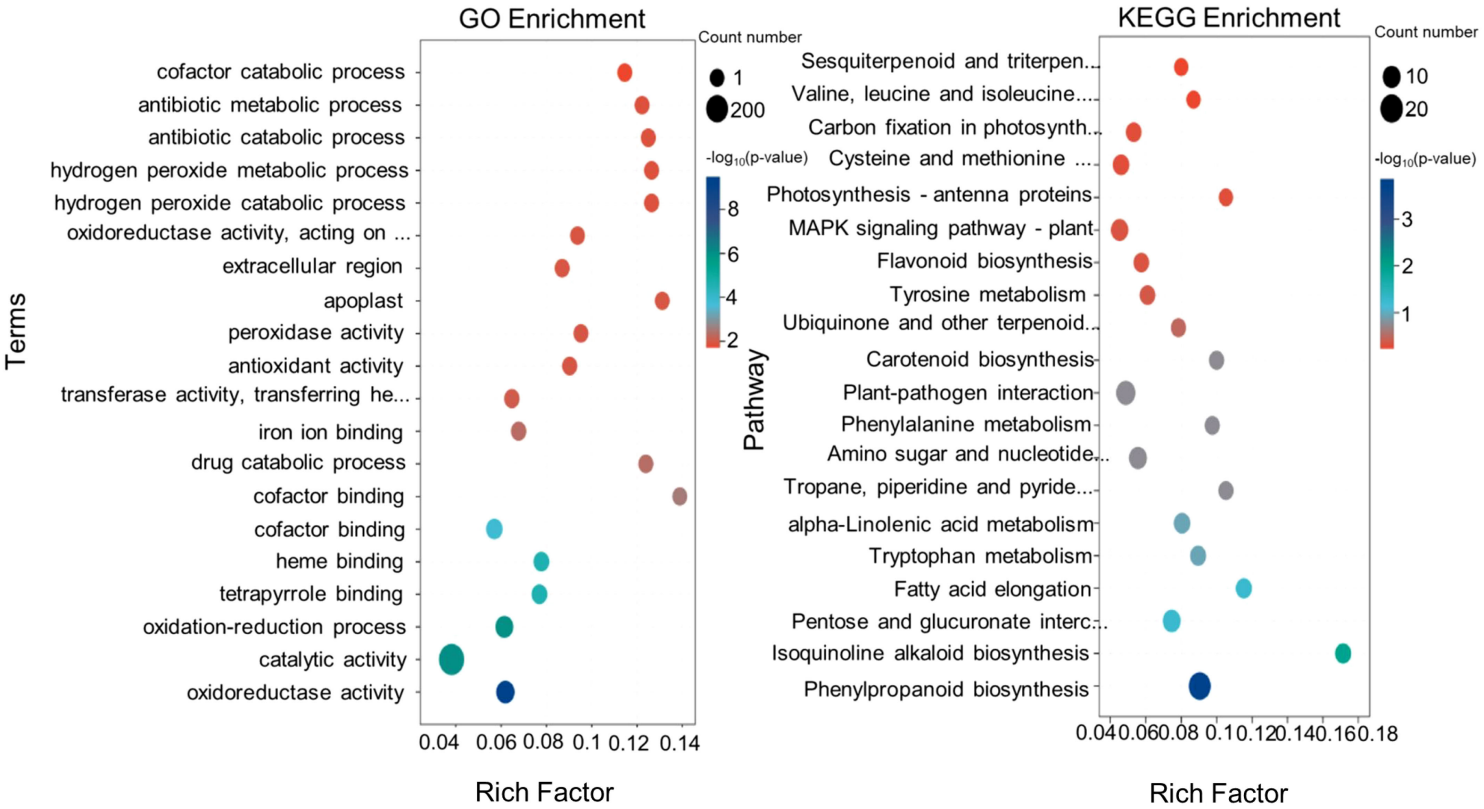
The GO and KEGG enrichment analyses of DEGs. **(A)** Bubble diagram for GO enrichment analysis of differential genes. **(B)** Bubble diagram for KEGG enrichment analysis of differential genes.

Functional enrichment of DEGs in Tartary buckwheat roots of colonized and uncolonized *S. indica* was analyzed using GO and KEGG databases. To research the function of shared DEGs between the two groups, we annotated the DEGs and performed enrichment analysis using the Gene Ontology (GO) database. In **Figure 4A**, the top 10 GO terms with the smallest p-values that were significantly enriched in the biological process (BP), cellular component (CC), and molecular function (MF) categories are shown. The GO term with the smallest p-value in the BP category was oxidation-reduction process (GO:0055114), which had DEG counts of 88. the next 14 DEGs were enriched in drug catabolic process (GO:0042737). The items with the highest DEG numbers in MF were catalytic activity (GO:0003824), oxidoreductase activity (GO:0016491), and cofactor binding (GO:0048037), with 288, 99, and 56 DEGs, respectively. In contrast, the highest number of differential genes in CC was for membrane (GO: 0016020), with 75 DEGs. Subsequently, we compared the annotations of up-regulated and down-regulated DEGs in the GO database, and found that up-regulated DEGs were mainly enriched in the cofactor binding (GO:0048037), tetrapyrrole binding (GO:0046906) and heme binding (GO:0020037), and down-regulated genes were mainly enriched in catalytic activity (GO:0003824), oxidoreductase activity (GO:0016491), membrane (GO:0016020) (**Figures 4**, **5A**).

Kyoto Encyclopedia of Genes and Genomes (KEGG) pathway analysis showed that KEGG enrichment analysis identified the top 20 pathways with the lowest p-values (**Figure 5B**). Among them, there were 12 pathways with p-value <0.5 (**Supplementary Table S3**), which were Phenylpropanoid biosynthesis (KO00940), Isoquinoline alkaloid biosynthesis (KO00950), Fatty acid elongation (KO00062), Pentose and glucuronate interconversions (KO00040), Alpha-Linolenic acid metabolism (KO00592), Tyrosine Metabolism (KO00350), Piperidine and pyridine alkaloid biosynthesis (KO00960), Amino sugar and nucleotide sugar metabolism (KO00520), Carotenoid biosynthesis (KO00906), Plant-pathogen interaction (KO04626), Phenylalanine metabolism (KO00360), Ubiquinone and other terpenoid-quinone biosynthesis (KO00130). The results show that most DEGs were significantly enriched in phenylalanine biosynthesis (KO00940), Pentose and glucuronate interconversions (KO00130), Amino sugar and nucleotide sugar metabolism (KO00520) and other In the pathway. Among them, there was a significant enrichment of DEGs with up-regulated expression in the Phenylpropanoid biosynthesis pathway, Amino sugar and nucleotide sugar metabolism was the most significantly down-regulated.

### 3.4 Effect of *S. indica* on phenylpropanoid biosynthesis in Tartary buckwheat

**Figure 6:**
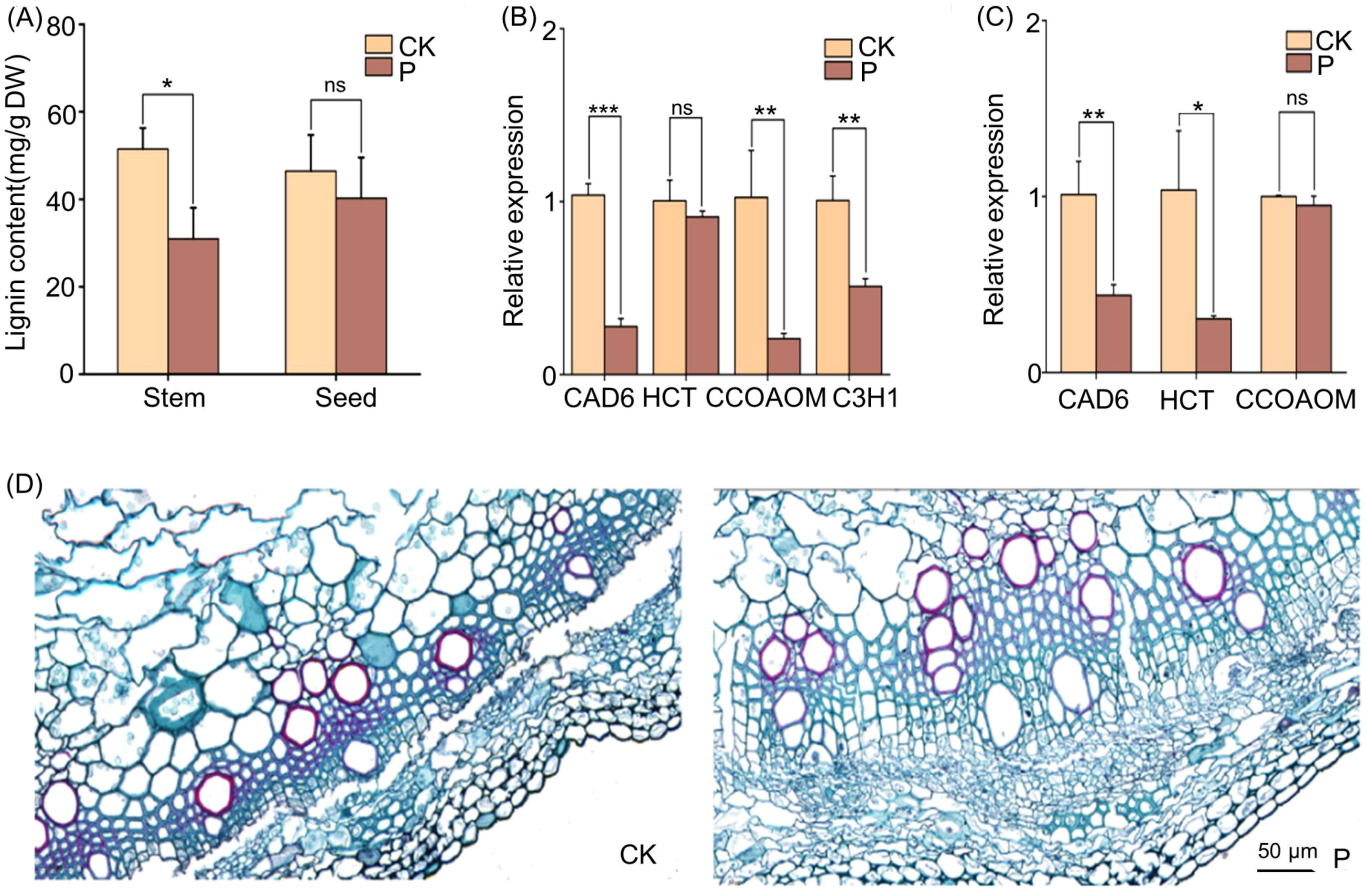
Lignin content and expression levels of genes of *S. indica* colonized (P) and non-colonized (CK) Tartary buckwheat. **(A)** Lignin content in stems and seeds of P and CK Tartary buckwheat. **(B)** Expression levels of genes related to the lignin biosynthesis pathway in P and CK Tartary buckwheat of seeds. **(C)** Expression levels of genes related to the lignin biosynthesis pathway in P and CK Tartary buckwheat of stems. **(D)** Histochemical analysis of stem cross-section lignin with Safranin-fixed green dyeing. Scale bar in **(D)** = 50 μm. Values are presented as mean ± SD (standard deviation) of three replicates. *p < 0.05; **p < 0.01; ***p < 0.001; ns p > 0.05.

The content of lignin in the stems and seeds of Tartary buckwheat was determined, and the results showed that the content of lignin in the stems and seeds of Tartary buckwheat was reduced after the addition of *S. indica* (**Figure 6A**). Key enzymes related to lignin synthesis were selected for further analysis, and the results of quantitative gene analysis showed that Tartary buckwheat seeds inoculated with *S. indica* showed reduced expression of the *FtHCT* gene, significantly lower expression of the *FtCCOAOM* and *FtC3H1* genes (p < 0.01), and a highly significant reduction in the expression of the *FtCAD6* gene (p < 0.001) compared with the control (**Figure 6B**). In *S. indica* colonized Tartary buckwheat stems, the expression of the *FtCCOAOM* gene was reduced, the expression of the *FtHCT* gene was significantly reduced (p < 0.05), and the expression of the *FtCAD6* gene was significantly reduced (p < 0.01) compared with the control (**Figure 6C**). It was further proved that the change of lignin gene expression level was basically consistent with the trend of content change. Next, the lignin staining of Tartary buckwheat stems was carried out with Safranin-fixed green dyeing, and the results showed that the color of lignin in the stems of Tartary buckwheat colonized with *S. indica* was significantly lighter than that in the stems of Tartary buckwheat uncolonized with *S. indica*, which indicated that the lignin content of Tartary buckwheat colonized with *S. indica* was reduced (**Figure 6D**).

### 3.5 Effect of *S. indica* on flavonoid biosynthesis in Tartary buckwheat

**Figure 7:**
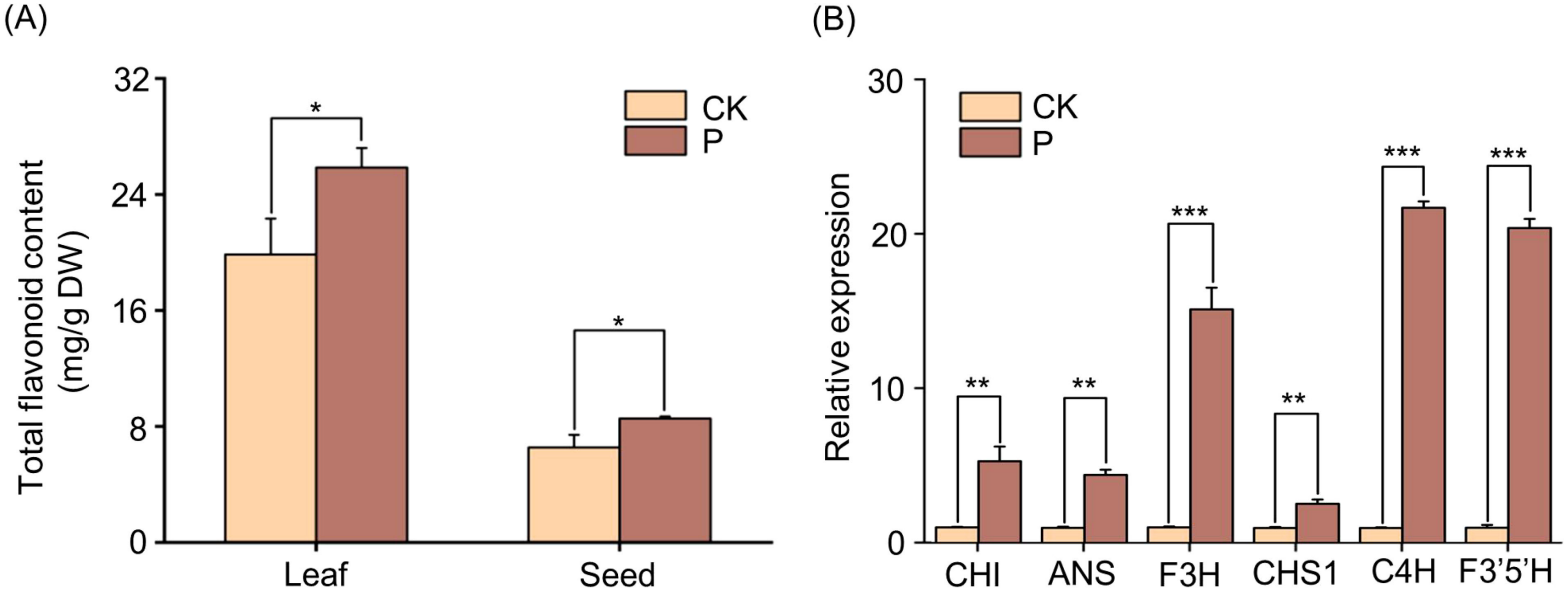
Flavonoids content and expression levels of genes of *S. indica* colonized (P) and non-colonized (CK) Tartary buckwheat. **(A)** Total flavonoids in leaves and seeds of P and CK Tartary buckwheat. **(B)** Expression levels of genes related to the flavonoid biosynthesis pathway in P and CK Tartary buckwheat seeds. Values are presented as mean ± SD (standard deviation) of three replicates. *p < 0.05; **p < 0.01; ***p < 0.001.

Further, the content of flavonoids in leaves and seeds of Tartary buckwheat was determined by *S. indica*, and the results showed that the content of flavonoids in leaves and seeds of Tartary buckwheat was significantly elevated by the addition of *S. indica* (**Figure 7A**). The key enzymes related to flavonoid synthesis were selected for further analysis, and the results of quantitative gene analysis showed that Tartary buckwheat seeds inoculated with *S. indica* showed significantly higher (p<0.01) expression of *FtCHI*, *FtANS* and *FtCHS1* genes, and highly significantly higher (p<0.001) expression levels of *FtF3H*, *FtC4H* and *FtF3’5’H* genes, as compared with the control (**Figure 7B**). The results indicated that the alteration of flavonoid gene expression levels in Tartary buckwheat colonized with *S. indica* was consistent with the trend of altered flavonoid content.

## 4 Discussion

Several researches have shown that *S. indica* can extensively colonize conductive plants (Mensah et al., 2020) and establish a symbiotic relationship with the plants, and it can significantly improve the yield and quality of the plants (Chen et al., 2022; Kundu et al., 2022; Solanki et al., 2023). It has been reported that *S. indica* significantly increased the yield of Tartary buckwheat under both drought and normal conditions (Zheng et al., 2023), which is consistent with this paper where *S. indica* which is consistent with this paper that *S. indica* increased the yield of Tartary buckwheat (**Figure 1**). However, current research on the effect of *S. indica* on Tartary buckwheat is mainly focused on phenotypes and other aspects. Research on the ways in which *S. indica* improves the seed quality of Tartary buckwheat is very limited, which makes the nutritional and economic value of *S. indica* on Tartary buckwheat overlooked.

**Figure 8:**
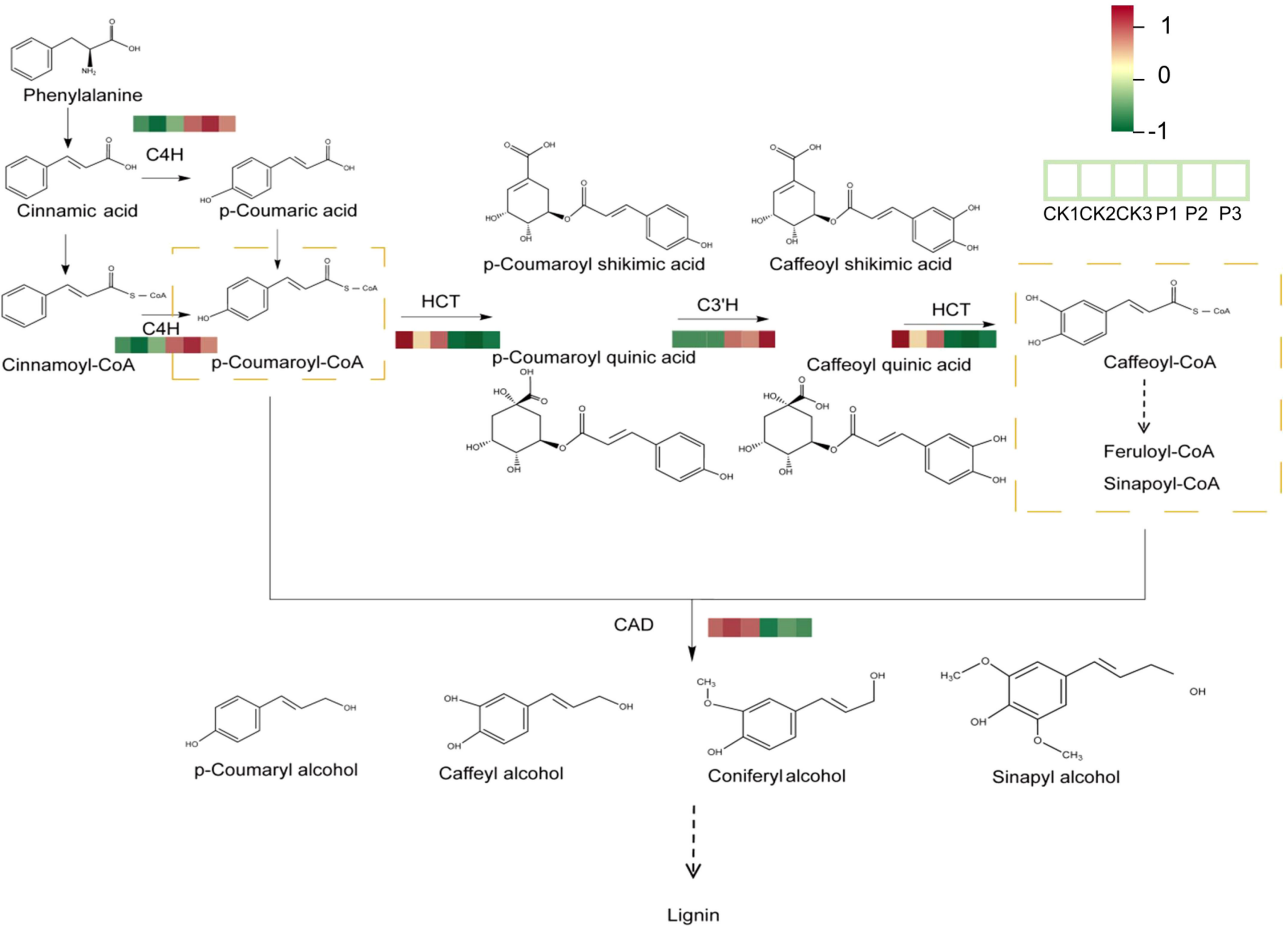
Lignin biosynthetic pathway involved in Tartary buckwheat the response to *S.indica*. Red and green indicate upregulation and downregulation, respectively. Intensity of the colors is proportional to the log_2_FC.

Lignin usually plays an important role in plant secondary walls (Geng et al., 2020). Its synthesis is mainly divided into three parts, the first of which is the deamination of phenylalanine to produce cinnamic acid, the second of which is the formation of lignin monomers through a series of hydroxylation, O-methylation, and reduction reactions, and the last part of which polymerizes the lignin monomers to form lignin (Liu et al., 2011). In the present research, we have further focused on the transcriptome analysis of the Phenylpropanoid biosynthesis pathway (**Figure 5B**). And found that cinnamyl-alcohol dehydrogenase (CAD), the key enzyme for the synthesis of lignin precursors such as p-Coumaryl alcohol, Caffeyl alcohol, Coniferyl alcohol, and Sinapyl alcohol, was basically down-regulated in roots (**Figure 8**). According to research, CAD is usually involved in the last step of lignin monomer synthesis, and the expression level of CAD gene is positively correlated with the total lignin content (Reddy et al., 2005; Tao et al., 2009; Shi et al., 2022). Caffeoyl-CoA O-methyltransferase (CoAOMT), CAD, and shikimate O-hydroxycinnamoyl transferase (HCT) are key enzymes for lignin biosynthesis. In this experiment, the relationship between lignin content and the expression of key enzyme genes *FtCoAOMT, FtCAD* and *FtHCT* was determined, and it was found that the lignin content was basically consistent with the trend of gene expression (**Figures 6A, B**). Therefore, it is hypothesized that *S. indica* may have reduced the lignin content of Tartary buckwheat seeds, and further experiments on functional expression are needed to show the causal relationship between changes in gene expression and phenotypic results. Tartary buckwheat is a food and herbal medicinal crop, due to its thick, hard and completely closed shells, the increase in lignin content increases the hardness of the shells of Tartary buckwheat, thus down-regulating the expression of genes in the lignin synthesis pathway and reducing lignin synthesis, which may lead to the thinning of the shell thickness and the decrease in lignin content. may lead to thinner hull thickness, thereby promoting fruit dehiscence (Li et al., 2020). Tartary buckwheat faces production and processing challenges of difficult hull removal, and *S. indica* makes the lignin content in the seeds of Tartary buckwheat decrease, which is hypothesized to facilitate the dehiscence of Tartary buckwheat. However, the thickness of hulls is closely related to the ratio of lignin to cellulose, and the species and proportion of lignin (Song et al., 2019; Yang et al., 2024). It is necessary to pay further attention to the content of cellulose in the hulls of Tartary buckwheat and the expression of genes, and the proportion of different lignin species in the hulls, to determine the effect of *S. indica* on the hulling of Tartary buckwheat.

**Figure 9:**
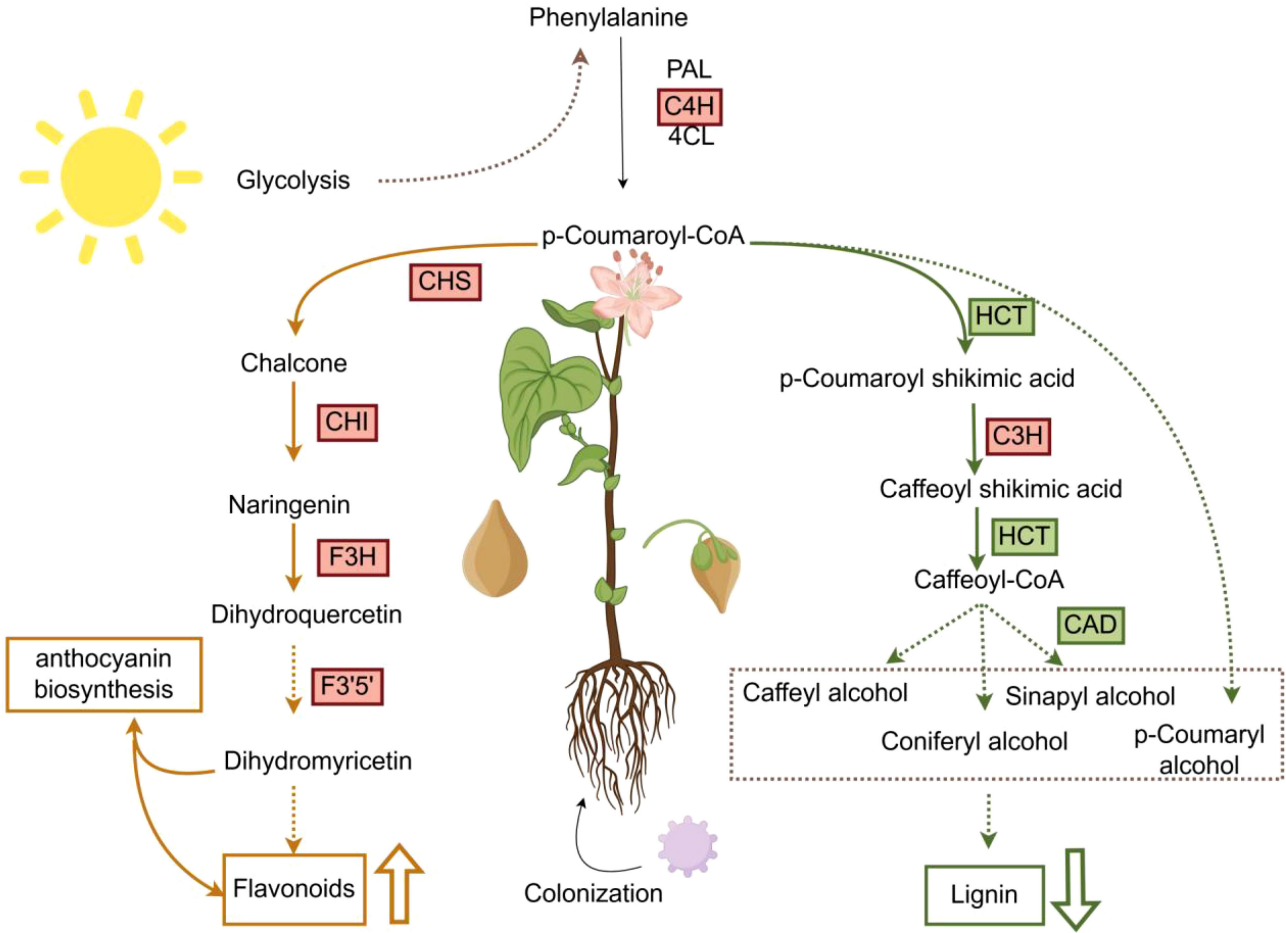
Summary diagram for the promotive effects of *S. indica* on tartarybuckwheat growth. Yellow and green arrows indicate the flavonoid biosynthesis pathway and lignin biosynthesis pathway, respectively. Red and green boxes indicate up and down regulation of gene expression.

Phenylpropanoid biosynthesis pathway includes lignin biosynthesis pathway and flavonoid biosynthesis pathway. In this research, we found that the lignin content in the phenylpropanoid biosynthesis pathway was reduced in the Tartary buckwheat of *S. indica* colonized compared with that of *S. indica* uncolonized. However, p-Coumaroyl-CoA, p-Coumaric acid p-Coumaric acid and other substances to rise. These substances are precursors for the synthesis of lignin and flavonoids, and it is hypothesized that these substances may flow into the flavonoid biosynthesis pathway, leading to an increase in the flavonoid content of Tartary buckwheat colonized with *S. indica* (**Figure 9**) (Song et al., 2019). Therefore, in this experiment, we determined the flavonoid content of Tartary buckwheat content with the expression of the corresponding key enzyme genes such as *FtCHI, FtANS, FtCHS1, FtCHI, FtANS, FtCHS1*, and it was found that the content was up-regulated in a consistent relationship with the expression of the genes (**Figure 7**), which led to a significant increase in the flavonoid content of the leaves and seeds of Tartary buckwheat colonized with *S. indica.* While, flavonoid compounds include isoflavones, flavonols and anthocyanins, flavonoids etc. Flavonoids affect the growth and development of plants and also act as antioxidants or signaling molecules to help plants to cope with adverse environments (Wang et al., 2017; Andrade et al., 2018; Liu et al., 2022; Wu et al., 2023; Yu et al., 2024). In addition to this, flavonoids have beneficial effects on human health and help to prevent diseases such as cancer, cardiovascular diseases and diabetes (Havsteen, 2002; Zhang et al., 2012; Sebastian et al., 2017; Ragheb et al., 2020). Therefore, *S. indica* has a significant impact on the enhancement of nutritional value of Tartary buckwheat and also provides new ways to improve the quality of Tartary buckwheat.

The study revealed a notable enrichment of the phenylpropanoid biosynthetic route within the transcriptome. The creation of phenylpropanoids stands as a crucial secondary synthesis route, beginning with phenylalanine and evolving into ρ-coumaroyl-coenzyme A through the activity of essential enzymes like phenylalanine ammonia-lyase (PAL), cinnamate 4-hydroxylase (C4H), and 4-coumarate–CoA ligase (4CL), followed by the transformation of ρ-coumaroyl-coenzyme A, the initial substrate, into lignin, flavonoids, and various other biosynthesis routes (Garibay-Hernández et al., 2021; Tohge et al., 2017). Transcriptome analysis revealed that the Phenylpropanoid biosynthesis pathway in Tartary buckwheat colonized with *S. indica* was significantly enriched in the KEGG database, leading to a series of alterations in lignin and flavonoid biosynthesis. A similar phenomenon has been found in several studies, where *S. indica* colonization in different plants increases the activity of genes within the Phenylpropanoid biosynthesis, leading to the up-regulation of lignin-related genes in some plants. This is different from the phenomenon observed in this study of lignin gene expression and content in Tartary buckwheat buckwheat (Bajaj et al., 2018; Li et al., 2023b). Whereas a large number of studies have also shown that *S. indica* increases the flavonoid content of the plant under abiotic stress, which is also different from the results of the present study, suggesting that *S. indica* may be able to increase the flavonoid content of Tartary buckwheat both under abiotic stress and under normal conditions. Furthermore, it has been shown that changes in key enzymes involved in the synthesis of lignin and flavonoids in the Phenylpropanoid biosynthesis pathway directly or indirectly lead to changes in the metabolic equilibrium between the lignin and flavonoid biosynthesis pathways (Nabavi et al., 2020; Wang et al., 2024). Further suggesting that the colonization of Tartary buckwheat by *S. indica* leads to a change in the control of lignin and flavonoids, resulting in Tartary buckwheat sacrificing part of the structural defense against lignin synthesis and prioritizing the chemical defense against flavonoid synthesis and nutrient growth. In addition, our data represent only one period of time during which *S. indica* altered lignin and flavonoid metabolism in Tartary buckwheat, and more sampling sites may be needed to clarify whether changes in these substances are species-specific or time-related. In conclusion, further attention needs to be paid to the mechanism of flavonoid accumulation in order to explain the enhancement of the nutritional value of Tartary buckwheat by woad from a more comprehensive point of view.

## 5 Conclusion

In summary, our results showed that *S. indica* colonization on Tartary buckwheat significantly increased the yield of aboveground parts of Tartary buckwheat along with roots. Further transcriptome analysis further revealed that phenylpropanoid biosynthetic was highly enriched in the KEGG database. Studies have focused on lignin and flavonoid synthesis pathways downstream of phenylacetone biosynthesis. *S. indica* was found to promote flavonoid synthesis in buckwheat seeds in addition to inhibiting lignin content in Tartary buckwheat seeds, and the effect of *S. indica* on key enzymes of lignin and flavonoid synthesis in Tartary buckwheat was further explored in terms of molecular mechanisms. The results showed that *S. indica* could not only reduce the lignin content but also increase the flavonoid content of Tartary buckwheat seeds, which provided new clues and potential methods to improve the nutritional and economic value of Tartary buckwheat seeds.

## Data Availability

The raw data supporting the conclusions of this article will be made available by the authors, without undue reservation.
