# Supplementary material for: Transcriptome analysis reveals changes in lignin and flavonoid biosynthesis in Serendipita indica colonized Tartary buckwheat
Source: Front Plant Sci. 2025 Jun 5;16:1595781. doi: 10.3389/fpls.2025.1595781 (PMC12176773; doi:10.3389/fpls.2025.1595781)
Supplement: Supplementary file 1 [file DataSheet1.docx]

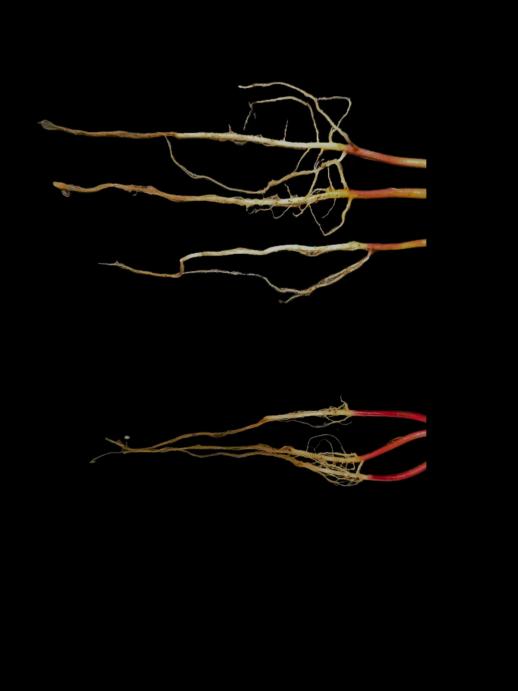


CK

P

**Supplementary figure 1**

**Analysis of phenotypes of roots of plants of Tartary buckwheat inoculated with *S. indica.***

P


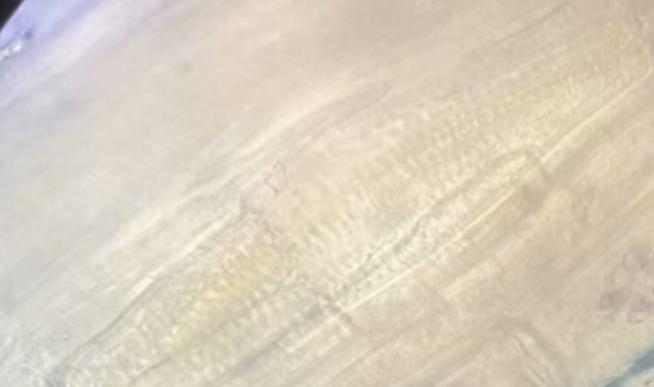

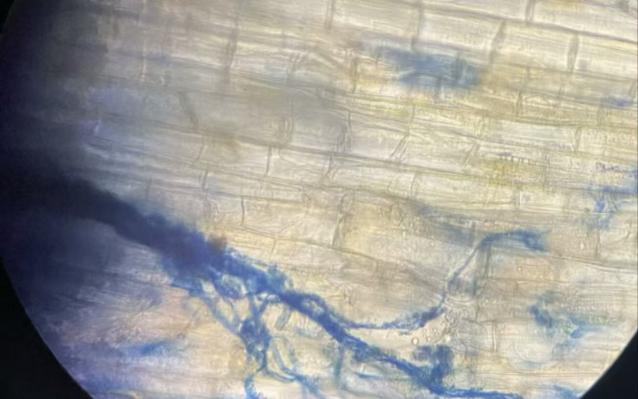


B

A

C

**Supplementary figure 2**

***S. indica*-colonized (A) and non-colonized (B) detection result.**
